# Knowledge and Attitude Regarding the Use of Sun Protection to Prevent Adverse Laser Events Among the General Population in Saudi Arabia: A Cross-Sectional Study

**DOI:** 10.7759/cureus.50157

**Published:** 2023-12-08

**Authors:** Ghadeer E Alamri, Mohammed F Bondagji, Lina I Kinkar, Eid A Almasoudi, Sarah M Fageeh, Lama G Asiri, Emad Bahashwan

**Affiliations:** 1 Medicine and Surgery, College of Medicine, Umm Al-Qura University (UQU), Makkah, SAU; 2 Faculty of Medicine, University of Jeddah, Jeddah, SAU; 3 Medicine and Surgery, College of Medicine, King Khalid University, Abha, SAU; 4 Internal Medicine, Dermatology, Faculty of Medicine, University of Bisha, Bisha, SAU

**Keywords:** saudi arabia, knowledge, awareness, laser treatment, sunscreens, sun protection, sun exposure

## Abstract

Introduction

Prolonged sun exposure has been linked with the development of numerous medical and dermatological complications, such as skin cancer. Photoprotection can help reduce ultraviolet radiation (UVR)-induced skin damage and skin cancer. This study aims to assess the knowledge about and attitude toward the use of sun protection to prevent laser adverse events among the general population in Saudi Arabia.

Methodology

This is a cross-sectional, analytical, community-based study carried out among the general population (sunscreen users) in Saudi Arabia. A total of 600 participants were enrolled in the study. Data were collected using a validated online self-administered questionnaire using Google Forms. Data were analyzed using IBM SPSS Statistics for Windows, Version 21.0 (IBM Corp., Armonk, NY).

Results

A total of 600 sunscreen users were enrolled in this study, with an overall poor knowledge rate of 471 (78.5%) regarding the use of sun protection methods. Their ages ranged from 18 years to >55 years. The majority of them were females (537, 89.5%), had Saudi Nationality (533, 88.8%), and had skin type III (313, 52.2%). Almost all the participants (491, 81.9%) had undergone laser treatment before; the most reported reason was hair removal (522, 87%). In addition, 267 (44.5%) participants used sunscreens five to six times a week, with 440 (73.3%) also using sunglasses. Notably, only 91 (15.2%) of the study participants were aware that sunscreen covers UVA and UVB, and 34 (5.7%) knew that PA+++ is used in sunscreen. A total of 149 (24.8%) reported that sunscreen should be applied 20 to 30 minutes before sun exposure, while 153 (25.5%) stated that it should be reapplied every two hours. Moreover, 484 (80.7%) participants reported using topical steroid application after laser treatment. The results also showed that young participants (*P *= 0.001), single participants (*P *= 0.001), post-graduate participants (*P *= 0.010), students rather than the unemployed group (*P *= 0.002), and those who used sunscreens five to six times per week compared to those who never used sunscreens (*P *= 0.001) demonstrated an overall good knowledge about sunscreens and laser treatment.

Conclusions

The study showed poor knowledge among the participants regarding the use of sun protection to prevent adverse laser events. Therefore, an increase in awareness among the general public about the protection through campaigns is highly recommended.

## Introduction

Ultraviolet radiation (UVR) is among the most influential environmental variables in the human body [1]. UVR improves health by lowering stress, enhancing mental activity, activating vitamin D3, and curing dermatological problems [2]. Conversely, extensive exposure to UVR has been linked to an increased risk of developing skin cancer and other forms of skin damage. It may also lead to sunburn, skin discoloration, and accelerated aging [3]. Photoprotection is essential for skin health, post-inflammatory hyperpigmentation (PIH) reduction, photoaging, and photocarcinogenesis prevention. Avoiding the sun, finding shade, using photoprotective clothing, wearing wide-brimmed hats and sunglasses, and using broad-spectrum sunscreens are all photoprotective strategies [4].

Lasers have completely changed the field of cosmetic dermatology by making it easier and safer to treat a wide range of skin diseases. Treatment options for a wide range of skin problems are now available, for example, benign vascular and pigmented birthmarks, tattoos, hypertrophic scars, and keloids [5]. Laser treatment may also cause unwanted side effects such as PIH, blisters, pain, crusting, hypopigmentation, purpura, dermatitis, and atrophic scarring [6]. Of the list of possible adverse effects, PIH is the most common. While more common in those with darker skin tones, PIH can affect people of any age and skin color. Inflammatory recurrence and/or sun exposure might make it much worse [7]. Treatment of PIH as soon as possible is vital for its resolution and for preventing additional darkening [8].

Numerous sun protection initiatives have been created in many Western countries to raise public awareness about the risks of sun exposure and promote the use of sun protection methods. However, sun protection compliance remains inadequate. There is limited research on this topic in Saudi Arabia [9]. Our study aims to assess the knowledge about and attitudes toward the use of sun protection to prevent adverse laser treatment events among the general population in Saudi Arabia.

## Materials and methods

Study design

This is a population-based cross-sectional study using a self-report questionnaire distributed through social media platforms with randomized sampling among the general population of Saudi Arabia between October and November 2022. Ethical approval was obtained from the Biomedical Ethics Committee of the Faculty of Medicine at Umm Al-Qura University (UQU), Makkah Al-Mukarramah, Saudi Arabia (approval number HAPO-02-K-012-2023-04-1599).

Study population

This study included males and females aged over 18 years who had previously undergone laser treatment. We excluded non-Arabic speakers.

Data collection and instrument survey

A reliable, simple, understandable, Arabic-based questionnaire was designed in Google Forms. The method protected participants' privacy and was a self-reported questionnaire. The questionnaire was distributed electronically via social media to the target population. We grouped the items of the questionnaire into five main sections: Section 1 - consent form; Section 2 - sociodemographic characteristics (age, gender, level of education, etc.); Section 3 - use of laser and history of laser treatment; Section 4 - questions that assess participants' behavior toward and knowledge of sun protection; and Section 5 - assessment of participants' attitude toward and knowledge of sun protection to prevent adverse laser treatment events. The study questionnaire was reviewed by two experts and validated via a pilot study on 20 persons.

Sample size and sampling technique

The minimum sample size required for this study was calculated by OpenEpi Version 3.0 in consideration of the following. The population size was about 8,325,304 individuals (according to the General Authority for Statistics), a confidence interval (CI) level set at 95%, and an anticipated frequency of 50%. The sample size was calculated to be 385 participants. The total sample size was 400 participants in case of dropout. However, the final sample size was 600.

Statistical analysis

Data were collected and reviewed manually and then analyzed using IBM SPSS Statistics for Windows, Version 21.0 (IBM Corp., Armonk, NY. All statistical methods used were two-tailed, with an alpha level of 0.05. Findings were considered significant if the *P*-value was less than or equal to 0.05. Overall knowledge levels regarding sunscreens and laser treatment were assessed by summing up discrete scores for different correct knowledge items. The overall knowledge score was categorized as low if the participant’s score was less than 60% of the overall score. Good awareness was defined by a score of 60% or more of the overall score. 

Descriptive analysis used frequency distributions and percentages for study variables, including participants’ data, history of laser treatment, as well as associated complications, sun exposure, and behaviors. Participants' knowledge about sunscreens and their role in laser treatment was tabulated, and participants’ overall knowledge and practices were plotted. Cross-tabulations showing the distribution of participants’ overall knowledge level by their data and other factors used Pearson’s chi-square test for significance and exact probability test if there were small frequency distributions.

## Results

A total of 600 sunscreen users in Saudi Arabia met the inclusion criteria and were included in the study. The ages ranged from 18 to over 55 years, with a mean age of 27.8 ± 13.6 years. Of the respondents, 537 (89.5%) were females and 533 (88.8%) were Saudi. By region, 209 (34.8%) participants were from the central region, 139 (23.2%) from the eastern region, and 104 (17.3%) from the western region. Considering skin type, 313 (52.2%) had light brown skin, 163 (27.2%) had white skin, and 95 (15.8%) had moderate brown skin, as detailed in Table 1.

**Table 1:** Personal characteristics of study participants in Saudi Arabia.

Personal data	n	%
Age in years		
18-25	220	36.7
26-35	179	29.8
36-45	115	19.2
46-55	69	11.5
>55	17	2.8
Gender		
Male	63	10.5
Female	537	89.5
Region		
Central	209	34.8
Northern	54	9.0
Eastern	139	23.2
Western	104	17.3
Southern	94	15.7
Marital status		
Single	267	44.5
Married	312	52.0
Divorced/widow	21	3.5
Educational level		
Secondary/below	98	16.3
Diploma	74	12.3
Bachelor	384	64.0
Post-graduate	44	7.3
Employment		
Unemployed	175	29.2
Student	184	30.7
Employed	199	33.2
Free works	42	7.0
Nationality		
Saudi	533	88.8
Non-Saudi	67	11.2
Skin type		
Pale white skin	20	3.3
White skin	163	27.2
Light-brown skin	313	52.2
Moderate brown skin	95	15.8
Dark-brown skin	8	1.3
Black skin	1	2

As for laser treatment and associated complications, 168 (28%) underwent laser treatment one to three times in the last year, and 160 (26.7%) had undergone it more than 10 times. Regarding the reasons for laser treatment, the most commonly reported was for hair removal (522, 87%). Specifically, out of 174 (29%) laser users, complications were experienced, including erythema in 83 (47.7%) and skin burns in 64 (36.8%), as detailed in Table 2.

**Table 2:** Laser treatment and associated complications among study participants in Saudi Arabia.

Laser treatment	n	%
Times of laser treatment		
1-3 times	168	28.0
4-6 times	163	27.2
7-9 times	109	18.2
≥10 times	160	26.7
Do you know the name of laser?		
Yes	286	47.7
No	314	52.3
Reasons for using laser treatment		
Hair removal	522	87.0
Acne	62	10.3
Dark spots	48	8.0
Melasma	25	4.2
Freckles	15	2.5
Redness	12	2.0
Scar	9	1.5
Others	9	1.5
Wrinkles	8	1.3
Experienced complications after laser treatment?		
Yes	174	29.0
No	426	71.0
Type of complications (*n *= 174)		
Erythema	83	47.7
Skin burn	64	36.8
Hyperpigmentation	36	20.7
Scar	21	12.1
Others	11	6.3

The study also assessed behaviors and sun exposure among sunscreen users and revealed that 331 (55.2%) of study participants were exposed to the sun for less than 30 minutes, 67 (11.2%) had a history of sunburn for one time, and 56 (9.3%) had for two to three times. A total of 437 (72.8%) used sunscreens and 267 (44.5%) did this five to six times per week. Regarding methods of dealing with sun rays, 522 (87%) participants stayed in the shade, 440 (73.3%) wore sunglasses, 243 (40.5%) wore brimmed hats, and only 126 (21%) used an umbrella, as detailed in Table 3.

**Table 3:** Behaviors and sun exposure among study participants in Saudi Arabia.

Sun exposure	n	%
Duration of sun exposure		
<30 minutes	331	55.2
30 minutes	186	31.0
2 hours or more	83	13.8
History of sunburn in one year		
Never	477	79.5
1 time	67	11.2
2-3 times	56	9.3
Application of sunscreen products		
Never	163	27.2
2-3 times per month	76	12.7
2-4 times/week	94	15.7
5-6 times/week	267	44.5
Wearing a brimmed hat		
Never	357	59.5
2-3 times/month	41	6.8
2-4 times/week	46	7.7
5-6 times/week	156	26.0
Staying in the shade		
Never	78	13.0
2-3 times/month	83	13.8
2-4 times/week	98	16.3
5-6 times/week	341	56.8
Usage of an umbrella		
Never	474	79.0
2-3 times/month	31	5.2
2-4 times/week	24	4.0
5-6 times/week	71	11.8
Usage of sun glasses		
Never	160	26.7
2-3 times/month	72	12.0
2-4 times/week	81	13.5
5-6 times/week	287	47.8

Concerning knowledge of the use of sun protection to prevent adverse laser treatment events, nearly 91 (15.2%) of the study participants knew that sunscreen covers UVA and UVB, 263 (43.8%) knew that SPF 30-50 or more is used in sunscreen, and only 34 (5.7%) knew that PA+++ is used in sunscreen. As for the amount of sunscreen for the face, 221 (36.8%) mentioned that it should be a digit finger or two digits, and 205 (34.2%) stated that sunscreen should be used both at home and outdoors. A total of 149 (24.8%) reported that sunscreen should be applied 20 to 30 minutes before sun exposure, and 153 (25.5%) knew that it should be reapplied every two hours. Exactly 222 (37%) of the participants knew that sunscreen should be applied the next day after laser treatment, as detailed in Table 4.

**Table 4:** Knowledge about the use of sun protection to prevent adverse laser treatment events among the general population in Saudi Arabia. UVR, ultraviolet radiation; SPF, sun protection factor

Knowledge items	n	%
Sunscreen covers UVR		
UVA	97	16.2
UVB	34	5.7
Both	91	15.2
Don't know	378	63.0
SPF of sunscreen		
30-50 or more	263	43.8
<30	70	11.7
Don't know	267	44.5
PA of sunscreen		
PA+++	34	5.7
PA++	72	12.0
PA+	65	10.8
Don't know	429	71.5
Amount of sunscreen for face		
Digit finger or two digits	221	36.8
Small pea or half of one-digit finger	250	41.7
Don't know	129	21.5
When to apply sunscreen		
At home and outdoor	205	34.2
Outdoor only	363	60.5
At home only	32	5.3
Sunscreen application prior to sun exposure		
20-30 minutes before	149	24.8
5-10 minutes before	253	42.2
Immediately before	117	19.5
Don't know	81	13.5
Sunscreen reapplication		
Repeat every two hours	153	25.5
Repeat anytime	101	16.8
No repeat	346	57.7
When to apply sunscreen after laser treatment		
The next day after laser	222	37.0
2-3 days after laser	93	15.5
After the healing of the wound	160	26.7
Immediately after laser	125	20.8

**Figure 1:**
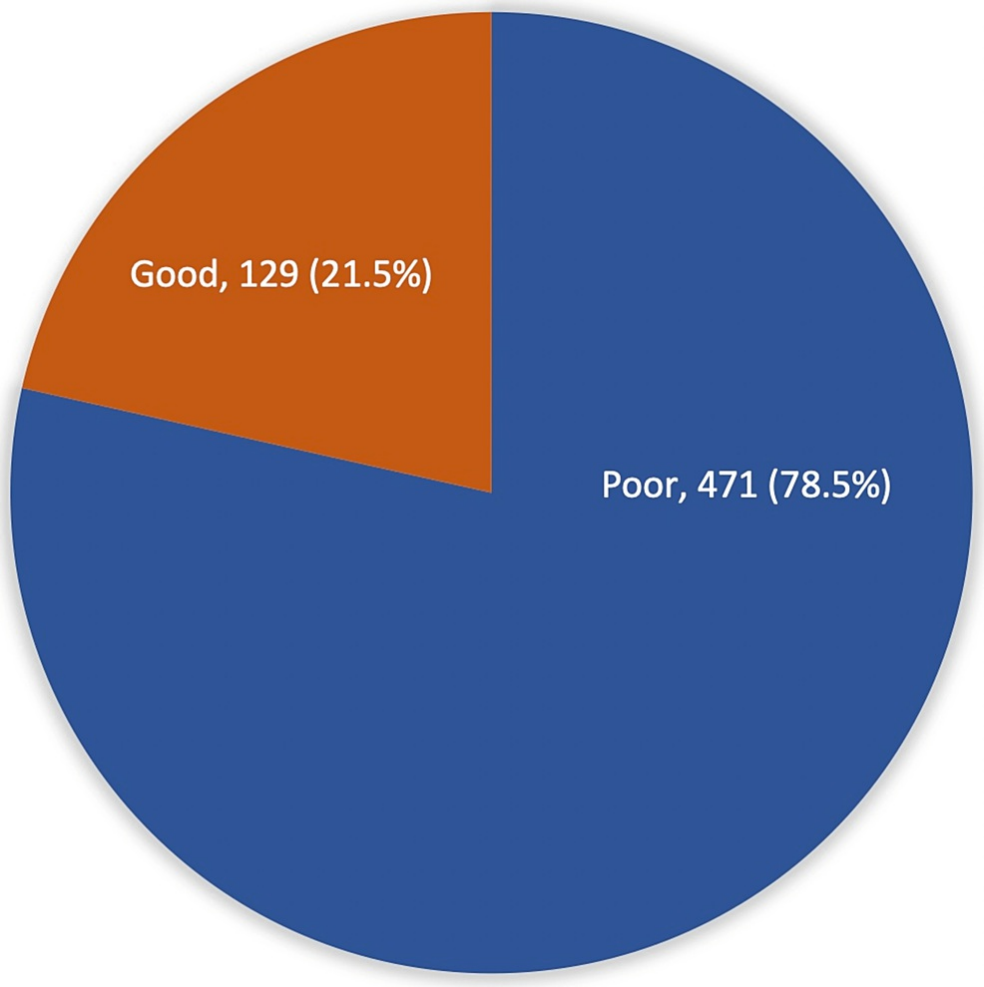
Overall participants' knowledge about the use of sun protection to prevent adverse laser treatment events in Saudi Arabia.

**Figure 2:**
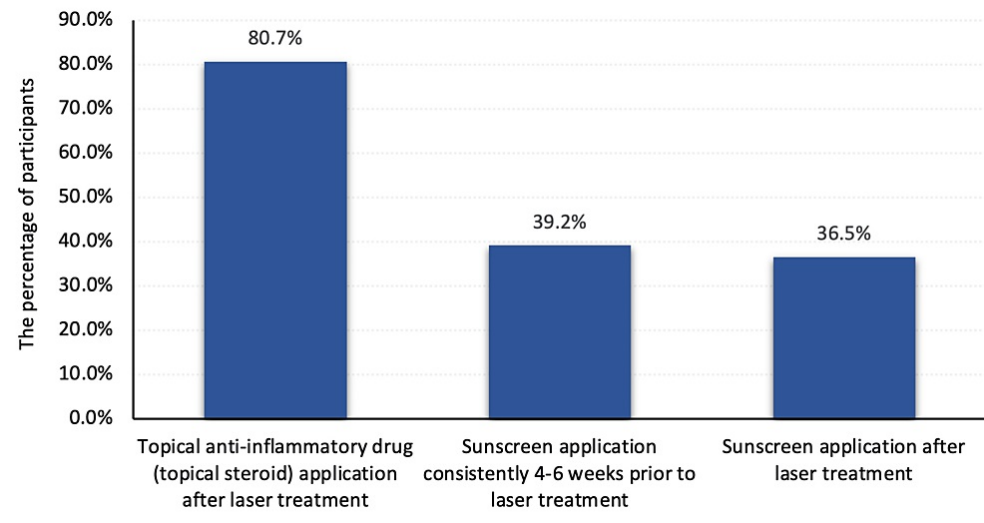
Practices and sunscreen use with laser treatment among study participants in Saudi Arabia.

Exactly 129 (21.5%) demonstrated an overall good knowledge about sunscreen use and laser treatment, while 471 (78.5%) had a poor knowledge level (Figure 1). Considering the practice and sunscreen use with laser treatment, a total of 484 (80.7%) reported using topical anti-inflammatory drug (topical steroid) application after laser treatment, 235 (39.2%) applied sunscreen four to six weeks before laser treatment, and 219 (36.5%) applied sunscreen after laser treatment (Figure 2).

The study showed that nearly 64 (29.1%) of young participants had an overall good knowledge level versus 2 (11.8%) of those aged more than 55 years with recorded statistical significance (*P *= 0.001). Also, 80 (30%) of single participants had a good knowledge level compared to 45 (14.4%) of married participants (*P *= 0.001). Good knowledge about sunscreens was detected among 12 (27.3%) post-graduate participants compared to 9 (9.2%) of those with low education (*P *= 0.010) and among 54 (29.3%) students in comparison to 22 (12.6%) of the unemployed group (*P *= 0.002). Likewise, 28 (33.7%) of those who were exposed to sun rays for two hours or more had good knowledge about sunscreens compared to 56 (16.9%) of those who were exposed less than 30 minutes (*P *= 0.002). In addition, 93 (34.8%) of those who used sunscreens five to six times a week had good knowledge compared to 6 (3.7%) of those who never used sunscreens (*P *= 0.001) (Table 5).

**Table 5:** Factors associated with participants' knowledge about sunscreens and laser treatment. ^*^Significant *P*-value. ^$^Exact probability test.

Factors		Overall knowledge level				*P*-value
		Poor		Good		
		n	%	n	%	
Age in years	18-25	156	70.9	64	29.1	0.001*
	26-35	139	77.7	40	22.3	
	36-45	97	84.3	18	15.7	
	46-55	64	92.8	5	7.2	
	>55	15	88.2	2	11.8	
Gender	Male	52	82.5	11	17.5	0.409
	Female	419	78.0	118	22.0	
Region	Central	174	83.3	35	16.7	0.098
	Northern	45	83.3	9	16.7	
	Eastern	99	71.2	40	28.8	
	Western	80	76.9	24	23.1	
	Southern	73	77.7	21	22.3	
Marital status	Single	187	70.0	80	30.0	0.001*
	Married	267	85.6	45	14.4	
	Divorced/widow	17	81.0	4	19.0	
Educational level	Secondary/below	89	90.8	9	9.2	0.010*
	Diploma	59	79.7	15	20.3	
	Bachelor	291	75.8	93	24.2	
	Post-graduate	32	72.7	12	27.3	
Employment	Unemployed	153	87.4	22	12.6	0.002*
	Student	130	70.7	54	29.3	
	Employed	156	78.4	43	21.6	
	Free works	32	76.2	10	23.8	
Skin type	Pale white skin	14	70.0	6	30.0	0.663^$^
	white skin	133	81.6	30	18.4	
	Light brown skin	245	78.3	68	21.7	
	Moderate brown skin	71	74.7	24	25.3	
	Dark brown skin	7	87.5	1	12.5	
	Black skin	1	100.0	0	0.0	
Times of laser treatment	1-3 times	137	81.5	31	18.5	0.190
	4-6 times	127	77.9	36	22.1	
	7-9 times	90	82.6	19	17.4	
	≥10 times	117	73.1	43	26.9	
Experienced complications after laser treatment?	Yes	131	75.3	43	24.7	0.221
	No	340	79.8	86	20.2	
Duration of sun exposure	<30 minutes	275	83.1	56	16.9	0.002*
	30 minutes	141	75.8	45	24.2	
	2 hours or more	55	66.3	28	33.7	
Application of sunscreen products	Never	157	96.3	6	3.7	0.001*
	2-3 times/month	69	90.8	7	9.2	
	2-4 times/week	71	75.5	23	24.5	
	5-6 times/week	174	65.2	93	34.8	

## Discussion

Sun UVR exposure is the principal source of vitamin D and a significant contributor to skin cancer [10]. One of the most important things that can be done to protect the skin from the hazardous effects of UVR is to use sunscreen. Sunscreen is a chemical substance designed to shield the skin from UV rays [11]. Skin cancer, including melanoma and nonmelanoma, can be prevented by wearing sunscreen [12,13].

This study aimed to assess knowledge of sun protection and its role in preventing adverse laser treatment events among the general population in Saudi Arabia. The study revealed that about one-fourth of the participants underwent laser treatment one to three times during the last year; another one-fourth underwent it four to six times; and the same percent underwent it more than 10 times. As for reasons for laser treatment, the most frequently reported were for hair removal (522, 87%). Less than one-third of laser users experienced complications. These included erythema, skin burns, hyperpigmentation, and scarring. Similar findings were reported by Al Hindi et al. [14] because hair removal is one of the most commonly reported cosmetic procedures in Saudi Arabia. Hammadi and El-Shereef [15] reported that about one-fourth of Saudi participants know about skin lasers for hair removal in Taif City. The reported complications were consistent with those most commonly reported in the literature related to laser skin treatment [16-19].

Regarding sunscreen use, this study showed that nearly three-fourths of those who underwent skin laser treatment used sunscreens 5 to 6 times per week, as reported by most participants. Al Jasser et al. [20] found that half of the Saudi students used sunscreen, with the main motivations being to avoid sunburns, dark spots, skin cancer, and general skin darkening. Another study revealed that the use of sunscreen among students at a Saudi university was about 139 (35%) [21]. The general population uses sunscreen less frequently than students (114, 8.3%) of the general population in Qassim [22]. About 629 (24%) of the general population in Saudi Arabia uses sunscreen according to a different cross-sectional study conducted in various regions [23].

Considering knowledge about sun protection and its role in preventing adverse effects of skin laser treatment, the current study showed that nearly one-fifth of the participants had a satisfactory knowledge level. In more detail, a few percentage of the study participants knew that sunscreen covers UVA and UVB, but less than half of them knew that SPF 30-50 or more is used in sunscreen. Only 34 (5.7%) knew that PA+++ is used in sunscreen. About one-third said that one or two fingers of sunscreen should be used for the face. They also knew that sunscreen should be used at home and outdoors. One-fourth reported that sunscreen should be applied 20-30 minutes before sun exposure, and the same percentage knew that it should be repeated every two hours. More than one-third of the participants knew that sunscreen should be applied the next day after laser treatment. Similar findings were reported by Almuqati et al. [8] among university students, where the majority of students (325, 64.9%) did not know about the sun protection factor of sunscreen products. The discomfort felt on the skin was the most commonly reported barrier to avoiding the use of sunscreen (204, 40.7%). Al Robaee [22] found that nearly 770 (56%) of participants reported awareness of the relationship between sunburn and skin cancer, and Al Ghamdi et al. [23] found that 1,449 (55.3%) of participants were aware of this relationship. Higher knowledge was reported among young participants, single participants, those with high education, and those who were exposed to the sun for a long duration.

Strength and limitations

This is the first study to be conducted in Saudi Arabia, to assess knowledge about and attitude toward the use of sun protection to prevent a post-laser adverse event among the general population.

The major limitation of this study is that results might be affected by recall bias because a standardized self-administered questionnaire was used to collect data.

## Conclusions

In conclusion, our study showed that 471 (78.5%) participants had poor knowledge about the use of sun protection to prevent adverse events after laser treatment; 91 (15.2%) participants knew that sunscreen covers UVA and UVB, and only 34 (5.7%) knew that PA+++ is used in sunscreen. Thus, we recommend an increase in awareness campaigns among the general public about sun protection.
